# D-galactose enhances Maillard reaction and antioxidant activity of arginine-derived products: experimental and molecular insights

**DOI:** 10.3389/fnut.2026.1924087

**Published:** 2026-08-28

**Authors:** Yanan Ding, Xinying Fan, Jingru Zhou, Yuqiong Wu, Pingfan Rao

**Affiliations:** 1 College of Tea and Food Science, Wuyi University, Wuyishan, China; 2 School of Food Science and Biotechnology, Zhejiang Gongshang University, Hangzhou, China; 3 SIBS-Zhejiang Gongshang University Joint Centre for Food and Nutrition Sciences, Zhejiang Gongshang University, Hangzhou, China; 4 Department of Neurology, Leiden University Medical Center, Leiden, Netherlands

**Keywords:** antioxidant activity, arginine, Maillard reaction, molecular docking, molecular dynamics simulation

## Abstract

**Background:**

Arginine-derived Maillard reaction products (MRPs) have shown promising antioxidant activities. However, how structurally distinct hexose reducing sugars, such as D-fructose and D-galactose, affect the formation and functional properties of L-arginine-derived MRPs remains unclear.

**Methods:**

Arginine-fructose (Arg-Fru) and arginine-galactose (Arg-Gal) model MRPs were prepared by heating at 100 °C for 0.5, 1, and 2 h. The reaction rate was analyzed by monitoring the reaction process and using a kinetic model, while substrate consumption and apparent melanoidin production were measured. Early non-covalent interactions were further investigated using molecular docking and 200 ns molecular dynamics (MD) simulations. Finally, functional differences were evaluated by chemical and intracellular antioxidant activities.

**Results:**

Compared with Arg-Fru, Arg-Gal showed a more great decrease in pH and higher A294 and A420 values under the same reaction conditions. Kinetic analysis showed that Arg-Gal had higher reaction rate constants. Meanwhile, Arg-Gal exhibited stronger UV-Vis absorption and fluorescence intensity, greater consumption of free amino groups and reducing sugars, and higher apparent melanoidin formation. Molecular docking results showed that D-galactose formed more hydrogen bonds with L-arginine than D-fructose; MD simulations further showed that the Arg-Gal system formed approximately 20 more hydrogen bonds on average, and the diffusion behavior of D-galactose was closer to that of L-arginine, suggesting stronger non-covalent association. Functionally, Arg-Gal showed stronger DPPH radical scavenging capacity, reducing power, and intracellular antioxidant activity than Arg-Fru.

**Conclusion:**

D-galactose may promote the Maillard reaction process by enhancing early non-covalent interactions with L-arginine and being associated with enhanced formation of intermediates and melanoidin-related substances, thereby enhancing the antioxidant activity of MRPs. These findings provide experimental and molecular insights into the role of sugar structure in modulating arginine-derived Maillard reaction systems.

## 1 Introduction

The Maillard reaction is a non-enzymatic browning process that occurs widely during food processing and thermal treatment, involving interactions between carbonyl compounds and amino groups. It generates a wide range of Maillard reaction products (MRPs), ranging from low- and intermediate-molecular-weight compounds to structurally complex, high-molecular-weight brown products known as melanoidins (1). These products contribute substantially to the color and flavor of thermally processed foods and have attracted increasing attention because of their potential biological activities, particularly antioxidant properties (2, 3). Recent studies have emphasized that the chemical composition, structural characteristics, and functional properties of melanoidins and other MRPs are strongly dependent on precursor composition and processing conditions (4). Therefore, elucidating how different reaction substrates influence MRP formation and functionality has become an important focus of food science research.

Sugar-amino acid model systems are widely used to investigate the reaction pathways, kinetics, and molecular mechanisms of the Maillard reaction under well-controlled conditions (5–7). Arginine, as a basic amino acid, exhibits considerable reactivity in the Maillard reaction, similar to lysine, and is closely associated with non-enzymatic glycation reactions (8, 9). In biological glycation, the guanidino group of protein-bound arginine residues is an important reaction site for reactive dicarbonyl compounds, such as methylglyoxal and glyoxal, leading to the formation of arginine-derived advanced glycation end products (AGEs) (10). Moreover, arginine is abundant in certain traditional Chinese medicinal materials. During the thermal processing of ginseng and other medicinal materials, arginine can react with reducing sugars to form MRPs, which have been reported to exhibit antioxidant activities in both chemical and cellular models (11). Previous studies have demonstrated that MRPs derived from arginine and fructose possess antioxidant activity and may help mitigate oxidative stress-related damage (12). Similarly, MRPs derived from arginine and glucose have been reported to effectively scavenge free radicals and protect immune cells against oxidative injury (13). Collectively, these findings indicate that arginine is a promising amino acid substrate for the preparation of bioactive MRPs.

The reactivity of reducing sugars varies markedly, leading to differences in Maillard reaction kinetics, browning intensity, molecular-weight distribution, volatile compound formation, and biological activity (14, 15). In general, pentoses exhibit higher Maillard reactivity than hexoses, whereas monosaccharides, such as glucose and fructose, are typically more reactive than reducing disaccharides (16, 17). Glucose is one of the most commonly used reducing sugar in Maillard reaction model systems, and arginine-glucose MRPs have been investigated previously (18, 19). The antioxidant properties of arginine-fructose MRPs have also been reported, whereas arginine-galactose MRPs remain comparatively less studied (12). D-Fructose and D-galactose are both reducing hexoses but differ in carbonyl type and molecular configuration. Accordingly, D-fructose was selected as a relatively well-studied reference substrate, whereas D-galactose was included as a less explored comparator to investigate whether structural differences between the two sugars influence their interactions with L-arginine and the subsequent formation and functionality of MRPs.

Although the general pathways of the Maillard reaction have been extensively investigated, the molecular events occurring at the initial stage of the reaction remain insufficiently understood. Before covalent bond formation, reducing sugars and amino acids may undergo transient non-covalent association involving molecular recognition and orientation, which may play a crucial role in determining the subsequent reaction pathway and kinetics. However, these transient molecular interactions are difficult to capture using conventional experimental approaches (20, 21). In recent years, molecular docking and molecular dynamics (MD) simulations have been increasingly used to investigate non-covalent interactions and conformational dynamics at the atomic level (22, 23). By revealing binding modes, hydrogen-bonding interactions, and conformational changes of reactants in solution, these computational approaches provide valuable mechanistic insights into the early stages of the Maillard reaction. Therefore, integrating molecular docking and MD simulations with experimental analyses may provide deeper mechanistic insight into how structural differences among reducing sugars affect their interactions with arginine, thereby contributing to differences in MRP formation and antioxidant activity.

Based on these considerations, two Maillard reaction model systems containing L-arginine and either D-fructose or D-galactose were established, and the corresponding MRPs were prepared by heating for 0.5, 1, and 2 h. The reaction kinetics, physicochemical characteristics, and antioxidant activities of the resulting MRPs were systematically compared. In parallel, molecular docking and MD simulations were performed to characterize the possible interaction modes and dynamic interactions between L-arginine and the two reducing sugars at the molecular level. By integrating experimental observations with computational analyses, this study aimed to elucidate how structural differences between D-fructose and D-galactose influence early-stage molecular interactions, reaction behavior, and the antioxidant properties of arginine-derived MRPs. The findings may provide mechanistic insights into the substrate-dependent formation of functional MRPs and support their potential development and application in food systems.

## 2 Materials and methods

### 2.1 Chemicals and reagents

L-Arginine, D-fructose, D-galactose, and 2,2-diphenyl-1-picrylhydrazyl (DPPH) were supplied by Sinopharm Chemical Reagent Co., Ltd. (Shanghai, China). 2,2’-Azobis(2-methylpropionamidine) dihydrochloride (AAPH) was purchased from Sigma-Aldrich (St. Louis, MO, USA). Dulbecco’s modified Eagle’s medium (DMEM), phosphate-buffered saline (PBS), Hanks’ balanced salt solution (HBSS), fetal bovine serum (FBS), and Penicillin-Streptomycin (100×) were obtained from Thermo Fisher Scientific. 3-(4,5-Dimethylthiazol-2-yl)-2,5-diphenyltetrazolium bromide (MTT) was purchased from MCE (Monmouth Junction, NJ, USA). Ultrapure water was produced using a Milli-Q water purification system (Millipore, Bedford, MA, USA). Unless otherwise specified, all other chemicals and solvents were of analytical grade and obtained from Sinopharm Chemical Reagent Co., Ltd. (Shanghai, China).

### 2.2 Instruments

XS105DU analytical balance (Mettler Toledo, Greifensee, Switzerland); FlexStation 3 microplate reader (Molecular Devices, San Jose, CA, USA); pH meter (Mettler Toledo, Greifensee, Switzerland); UV-5100 UV-Vis spectrophotometer (Hitachi, Tokyo, Japan); NU-8500 CO_2_ incubator (NuAire, Plymouth, MN, USA); XD-202 optical microscope (Nanjing Jiangnan Yongxin Optical Co., Ltd., Nanjing, China); and Milli-Q ultrapure water purification system (Millipore, Billerica, MA, USA).

### 2.3 Preparation of arginine-derived Maillard reaction products

Reaction mixtures were prepared by dissolving L-arginine and either D-fructose or D-galactose to a final concentration of 0.6 mol/L for each reactant. The mixtures were heated in a water bath at 100 °C for 0.5, 1, or 2 h, followed by immediate cooling in an ice-water bath to quench the reaction. The Maillard reaction products obtained from the fructose- and galactose-based model systems were designated as Arg-Fru and Arg-Gal, respectively.

### 2.4 Physicochemical characterization of Maillard reaction products

#### 2.4.1 Determination of pH

At predetermined heating intervals, the pH of the MRPs was measured using a pH meter, and the resulting values were used to evaluate the progression of the Maillard reaction.

#### 2.4.2 Determination of absorbance at 294 and 420 nm

Maillard reaction products were diluted 100-fold with ultrapure water. Absorbance was subsequently measured at 294 and 420 nm using a UV-Vis spectrophotometer, and the corresponding values were used to evaluate the formation of intermediate and final Maillard reaction products, respectively.

#### 2.4.3 UV-Vis spectral analysis

Before spectral analysis, the MRPs were diluted 100-fold with ultrapure water. UV-Vis absorption spectra were then acquired over the wavelength range of 250–700 nm.

#### 2.4.4 Fluorescence analysis

Before analysis, the MRPs were diluted 1000-fold with ultrapure water. Fluorescence intensity was subsequently determined using a FlexStation 3 microplate reader with excitation and emission wavelengths set at 347 and 415 nm, respectively.

### 2.5 Kinetic modeling of the Maillard reaction

Kinetic modeling was performed using a multi-criteria framework (Adj. R^2^, Reduced χ^2^, 95% CI, and residual diagnostics; Supplementary Figures 1–3 and Supplementary Table 1). For A294 and A420, the zero-order model was selected (Adj. R^2^ > 0.92) because it provided a better fit than the first-order alternative; for pH, the first-order model was selected because the zero-order model showed a poor fit (Adj. R^2^ = −0.100) (24). The corresponding kinetic equations are as follows:


$$A=A_{0}+K\,t$$

$$A=A_{0}\,\text{exp}\,\left(-K\,t\right)$$

where A_0_ and A represent the initial and time-dependent values of each parameter, respectively; K is the apparent reaction rate constant; and t is the reaction time.

### 2.6 Determination of free amino groups and reducing sugars

#### 2.6.1 Determination of reducing sugars

Reducing sugar contents of the MRPs were determined using the 3,5-dinitrosalicylic acid (DNS) assay (25, 26). Briefly, 750 μL of 100-fold diluted MRPs was mixed with 750 μL DNS reagent and heated in boiling water for 5 min. The reaction mixture was immediately cooled in an ice-water bath, followed by the addition of 750 μL ultrapure water. After thorough mixing, the absorbance was measured at 540 nm using a UV-Vis spectrophotometer. Glucose (0, 100, 200, 300, 400, 500, 600, 700, 800 μg/mL) was used as the standard for calibration, and the reducing sugar content was calculated based on the standard curve (*y* = 0.0029x−0.0028, R^2^ = 0.9933), where y represents absorbance and x represents glucose concentration. Since the DNS assay reflects the overall reducing capacity of sugars rather than the absolute concentration of individual sugar species, reducing sugar contents were calculated based on the glucose standard curve and expressed as glucose equivalents.

#### 2.6.2 Determination of free amino groups

1 mL of 100-fold diluted MRPs was mixed with 0.5 mL of phosphate buffer (pH 8.0) and 0.5 mL of 2% ninhydrin solution. The mixture was vortexed thoroughly and heated in a boiling water bath for 15 min. After cooling to room temperature, the reaction mixture was diluted to a final volume of 25 mL with distilled water and allowed to stand for 10 min. The absorbance was measured at 570 nm by a UV-Vis spectrophotometer using the reagent blank as the reference. L-Arginine (0, 0.2, 0.3, 0.4, 0.5, 0.6, 0.7, 0.8, 0.9, and 1.0 mg/mL) was used as the standard for calibration, and the free amino group content was calculated based on the standard curve *y* = 2.0289x (R^2^ = 0.9893), where y represents absorbance and x represents L-arginie concentration.

### 2.7 Quantification of apparent melanoidin concentration

The absorbance at 420 nm was used as an index of melanoidin formation. Because melanoidins are heterogeneous and structurally undefined MRPs, the calculated value represents an apparent concentration rather than an absolute molar concentration (27). The apparent melanoidin concentration was estimated based on the Beer-Lambert law:


A=ε×b×c

where A is the absorbance at 420 nm, ε is the apparent molar extinction coefficient (0.6 L/mmol/cm), b is the optical path length (cm), and c is the apparent concentration of melanoidins (mmol/L).

### 2.8 Molecular docking

The molecular structures of L-arginine (CID: 6322), D-galactose (CID: 2723872), and D-fructose (CID: 6036) were downloaded from the PubChem database and saved in SDF format. Each structure was subjected to energy minimization and hydrogen-atom addition using Open Babel (28). L-Arginine was designated as the flexible ligand, whereas D-galactose or D-fructose was assigned as the rigid receptor; the resulting structures were stored in PDBQT format. The docking grid box was manually defined to fully encompass the L-arginine molecule. Molecular docking was subsequently performed with AutoDock Vina 1.1.2, and the output binding modes were obtained in PDBQT format (29). The docking results from both systems were then converted to PDB format using AutoDockTools 1.5.6. Finally, Discovery Studio 2019 Client was employed to visualize and analyse intermolecular interactions (e.g., hydrogen bonds) in the best-scoring binding poses.

### 2.9 Molecular dynamics simulation

L-arginine molecule was constructed with Avogadro, and its topology files were generated using the pdb2gmx utility in GROMACS, while the molecular structures and corresponding topologies of D-fructose and D-galactose were prepared via the CHARMM-GUI web server (30–32). All MD simulations were performed using GROMACS 2021.3 with the CHARMM36m force field paired with the modified TIP3P water model (33, 34). Each initial system was assembled by randomly inserting 185 L-arginine molecules and 185 monosaccharides (either D-fructose or D-galactose) into a cubic water box with an edge length of 8 nm, and the system was rendered electrically neutral.

The simulation workflow proceeded sequentially. First, energy minimization was conducted with the steepest descent algorithm for 5000 steps to eliminate unfavorable steric clashes. This was followed by a 125 ps isothermal-isochoric (NVT) equilibration and a subsequent 500 ps isothermal-isobaric (NPT) equilibration, both performed with an integration time step of 1 fs. Finally, 200 ns production MD simulations were run with a 2 fs time step.

The system temperature was maintained at 373.15 K (100 °C, consistent with the experimental preparation conditions) across all simulation stages using the v-rescale thermostat with a temperature coupling relaxation time of 1.0 ps for both solute and solvent groups. The reference pressure was set to 1 bar (ref_*p*_ = 1.0 bar) with an isothermal compressibility of (4.5 × 10^–5^ bar^–1^); the C-rescale barostat was adopted for the NPT equilibration stage with a pressure coupling relaxation time (τ_*P*_) of 5.0 ps, and the Parrinello-Rahman barostat was applied in the production phase with an identical (τ_*P*_) value of 5.0 ps. Nonbonded interactions were treated under the Verlet cutoff scheme, where the neighbor list was updated every 20 simulation steps. Van der Waals interactions were calculated via the Cut-off formalism combined with the Force-switch modifier, with the van der Waals switching distance set to 1.0 nm, while the van der Waals cutoff (r_*vdw*_), neighbor list cutoff (r_*list*_), and direct-space electrostatic cutoff (r_*coulomb*_) were uniformly defined as 1.2 nm. Long-range Coulombic interactions were computed via the particle mesh Ewald (PME) method, and all covalent bonds involving hydrogen atoms were constrained using the LINCS algorithm. Hydrogen bonds between Arg and monosaccharides, as well as diffusion coefficients, were calculated using built-in GROMACS utilities, with analyses restricted to the trajectory segment from 100 ns to 200 ns. Uncertainty was estimated by block averaging, and hydrogen-bond numbers and diffusion coefficients are reported as the mean ± SD of the block values (35). Molecular visualization was implemented using Visual Molecular Dynamics (VMD).

### 2.10 Evaluation of chemical antioxidant activity

2,2-diphenyl-1-picrylhydrazyl radical scavenging activity and reducing power were determined according to previously reported methods with slight modifications (36).

#### 2.10.1 Determination of DPPH radical scavenging activity

The DPPH radical scavenging activity was determined according to a previously reported method with slight modifications. A DPPH solution (0.1 mg/mL) was freshly prepared in ethanol under dark conditions, and the MRP samples were diluted 100-fold before analysis. Briefly, 100 μL of the diluted MRP samples were added to a 96-well plate, followed by the addition of 100 μL of DPPH solution. The mixture was thoroughly mixed and recorded as A_1_. For sample background correction, 100 μL of ultrapure water was used instead of the DPPH solution and mixed with the sample, and the absorbance was recorded as A_2_. For reagent blank correction, 100 μL of ultrapure water was used instead of the sample and mixed with the DPPH solution, and the absorbance was recorded as A_3_. The mixtures were incubated at room temperature in the dark for 30 min, and the absorbance was measured at 517 nm using a FlexStation 3 microplate reader. Ascorbic acid (Vc) was used as the positive control. DPPH radical scavenging activity was calculated using the following formula:


$$DPPHfreeradicalscavengingrate\left(\%\right)=\left(1-\frac{A_{1}-A_{2}}{A_{3}}\right)\times 100$$

Where A_1_, A_2_ and A_3_ represent the absorbance values of the sample–DPPH mixture, sample blank, and DPPH blank, respectively.

#### 2.10.2 Determination of reducing power

The reducing power of MRPs was determined using the potassium ferricyanide reduction method. Briefly, 0.5 mL of 500-fold diluted MRP sample were mixed with 2.5 mL of 0.2 M sodium phosphate buffer (pH 6.6) and 2.5 mL of 1% (w/v) potassium ferricyanide solution. The mixture was incubated in a water bath at 50 °C for 20 min. After cooling to room temperature, 2.5 mL of 10% (w/v) trichloroacetic acid (TCA) solution was added, followed by centrifugation at 3000 rpm for 10 min. Subsequently, 2.5 mL of the supernatant was mixed with 2.5 mL of deionized water and 0.5 mL of 0.1% (w/v) ferric chloride solution. After incubation for 10 min, 200 μL of the final reaction mixture was transferred into a 96-well plate, and the absorbance was measured at 700 nm using a FlexStation 3 microplate reader. Vc was used as the positive control.

### 2.11 Cell culture

RAW 264.7 cells were obtained from the National Collection of Authenticated Cell Cultures (Shanghai, China). Cells were cultured in DMEM supplemented with 10% FBS, 100 U/mL penicillin, and 100 μg/mL streptomycin sulfate and maintained at 37 °C in a humidified atmosphere containing 5% CO_2_. Cells between passages 10 and 15 were used in all experiments, and cultures at approximately 80% confluence were selected for subsequent assays.

### 2.12 Cell viability assay

RAW 264.7 cells were seeded into 96-well plates at a density of 2 × 10^4^ cells/mL and allowed to attach for 24 h. The culture medium was then discarded and replaced with DMEM containing MRPs diluted 50-, 100-, or 200-fold. Cells cultured in DMEM without MRPs served as the blank control. Following a further 24 h incubation, 10 μL of MTT solution (5 mg/mL) was added to each well, and the plates were incubated at 37 °C for 2–4 h. The medium was carefully aspirated, after which 150 μL of DMSO was added to dissolve the crystals completely. The plates were gently shaken before measuring the absorbance at 570 nm using a FlexStation 3 microplate reader. Cell viability was calculated using the following equation:


$$Cellviability\left(\%\right)=\frac{\left(A_{2}-A_{0}\right)}{\left(A_{1}-A_{0}\right)}\times 100$$

where A_0_, A_1_, and A_2_ denote the absorbance values of the background blank, blank control, and sample-treated groups, respectively.

### 2.13 Cellular antioxidant activity assay

The intracellular antioxidant activity of MRPs was determined using the cellular antioxidant activity (CAA) assay (37). RAW 264.7 cells were seeded in 96-well plates at a density of 5 × 10^4^ cells/mL and cultured until approximately 80% confluence. After washing once with HBSS, the cells were incubated with 200 μL of basal DMEM containing 50 μM DCFH-DA and MRPs at final dilution factors of 50, 100, or 200 for 60 min. The cells were then washed once with HBSS, followed by treatment with 100 μL of AAPH solution (0.6 mM), while HBSS was added to the negative control. Fluorescence (Ex = 485 nm, Em = 538 nm) was recorded every 10 min for 60 min at 37 °C using a FlexStation 3 microplate reader. Quercetin was used as the positive control. CAA units were calculated using the following equation:


$$C\,A\,A\,u\,n\,i\,t\,s=100-\frac{\int S\,A}{\int C\,A}\times 100$$

where ∫SA and ∫CA represent the integrated areas under the fluorescence-time curves of the sample-treated and AAPH-treated control groups, respectively.

### 2.14 Statistical analysis

Results are presented as the mean ± standard deviation (SD) of three independent experiments (*n* = 3). Statistical analyses were conducted using SPSS Statistics 23.0 (SPSS Inc., Chicago, IL, USA). For time-dependent physicochemical and chemical antioxidant parameters, the effects of reaction system and heating time were evaluated using two-way analysis of variance (ANOVA), followed by Tukey’s *post hoc* test for multiple comparisons. For cell viability and CAA, two-way ANOVA was performed using MRPs and dilution factor as the two independent factors. Tukey’s *post hoc* test was subsequently used to compare different MRPs at the same dilution factor and different dilution factors within the same MRP sample. Kinetic parameters were obtained by regression fitting as described in section “2.5 Kinetic modeling of the Maillard reaction.” Statistical significance was defined as *P* < 0.05. Graphs were generated with GraphPad Prism 9.0 (GraphPad Software, San Diego, CA, USA). Molecular docking and MD simulation analyses were carried out as described in sections “2.8 Molecular docking” and “2.9 Molecular dynamics simulation,” respectively.

## 3 Results

### 3.1 Physicochemical changes during the Maillard reaction

**FIGURE 1:**
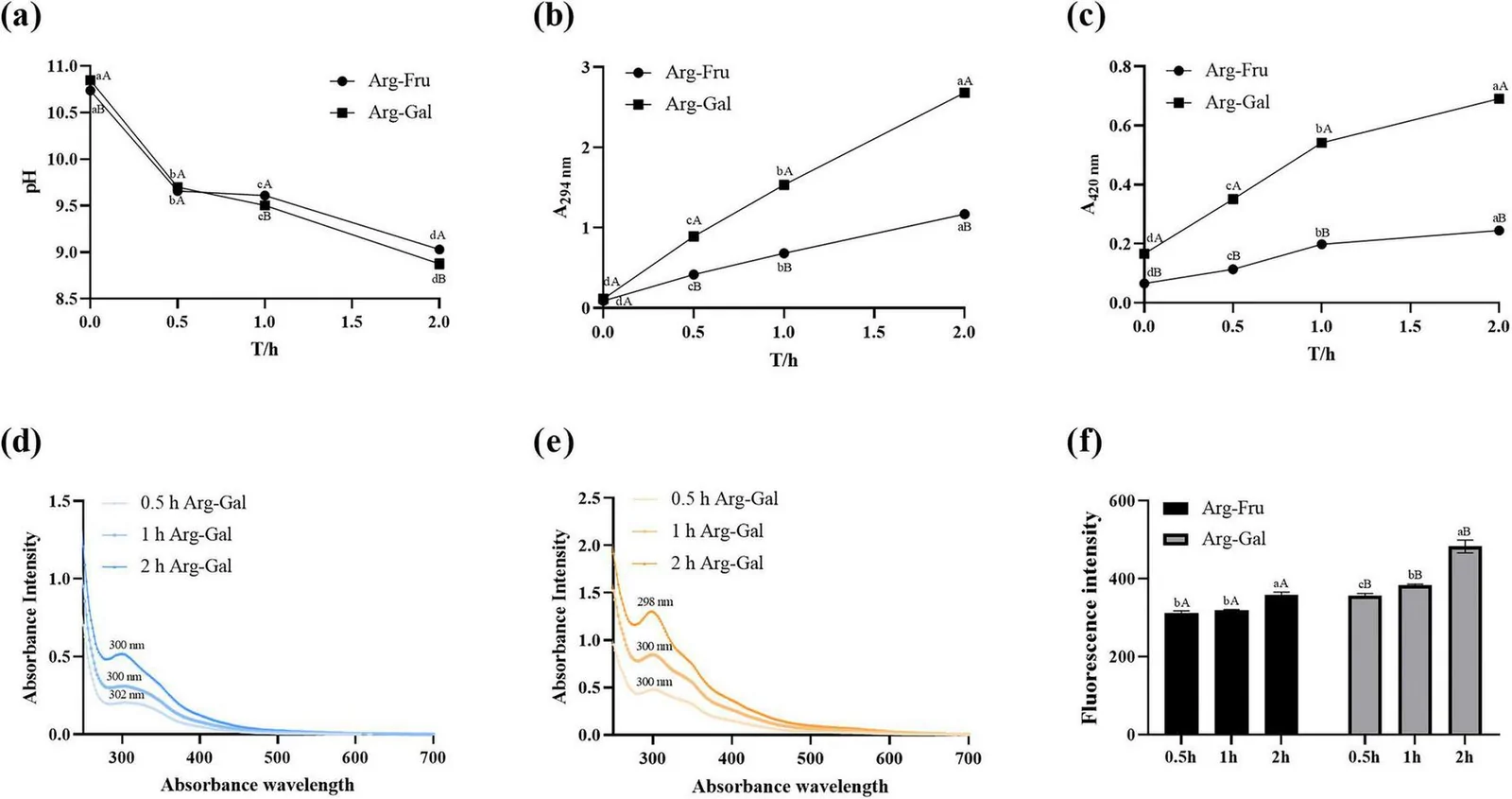
pH **(a)**, absorbance at 294 nm **(b)** and 420 nm **(c)** of MRPs; UV-Vis spectra comparison of MRPs: Arg-Fru **(d)** and Arg-Gal **(e)**; Fluorescence intensity of MRPs **(f)**. Different lowercase letters indicate statistically significant differences (*P* < 0.05) among different time points within the same MRPs, while different uppercase letters denote significant differences (*P* < 0.05) between different MRPs at the same time point.

Changes in the pH of the MRP systems during heating are shown in Figure 1a. At the beginning of the reaction, the two MRP systems showed similar pH values, which decreased significantly as heating proceeded. Notably, the pH of Arg-Gal was significantly lower than that of Arg-Fru after 1 and 2 h of heating. As shown in Figures 1b,c, the absorbance values of both systems at 294 nm and 420 nm increased significantly with heating time, reaching their maximum levels after 2 h. Specifically, Arg-Gal consistently exhibited higher absorbance at both 294 and 420 nm than Arg-Fru throughout the reaction period.

Figures 1d,e show the UV-Vis spectral profiles of Arg-Fru and Arg-Gal recorded over the wavelength range of 250–700 nm after heating for 0.5, 1, and 2 h. During the 2 h heating period, the UV-Vis absorbance of both systems increased progressively and reached the highest level at 2 h. The maximum absorption peak was observed at 298 nm for Arg-Fru and at 300 nm for Arg-Gal. Arg-Gal exhibited markedly higher UV-Vis absorbance than Arg-Fru. After 0.5, 1, and 2 h of heating, the maximum absorption intensities of Arg-Fru were 0.205, 0.310, and 0.519, respectively, whereas those of Arg-Gal were 0.478, 0.847, and 1.305, respectively.

As shown in Figure 1f, the fluorescence intensity of both MRPs increased with prolonged reaction heating. At 0.5, 1, and 2 h, Arg-Gal showed significantly higher fluorescence intensities than Arg-Fru, with values of 356.86 ± 5.63, 383.83 ± 2.61, 482.73 ± 16.69, respectively, compared with 312.53 ± 4.96, 319.37 ± 1.26, and 359.47 ± 6.23 for Arg-Fru.

### 3.2 Kinetic analysis of the Maillard reaction

The resulting kinetic parameters are summarized in Table 1. The rate constants for both A294 and A420 were higher in the Arg-Gal than in the Arg-Fru, with adjusted R^2^ values > 0.92, indicating a good fit to the zero-order kinetic model. Similarly, the acidification rate constant was higher in the Arg-Gal, with adjusted R^2^ values approximately in the range of 0.82–0.86, suggesting that the pH changes could be reasonably described by the first-order kinetic model. Overall, the Arg-Gal exhibited consistently faster reaction kinetics, suggesting higher Maillard reactivity than the Arg-Fru.

**TABLE 1:** Reaction kinetic parameters of the Arg-Fru and Arg-Gal.

Parameter	Group	A_0_	A_0_ 95% CI	K (h^–1^)	K 95% CI	Reduced χ ^2^	Adj. R^2^
pH	Arg-Fru	10.44642 ± 0.12533	[10.16673, 10.7277]	0.07988 ± 0.01124	[0.05476, 0.10527]	0.0746	0.8204
pH	Arg-Gal	10.54881 ± 0.12687	[10.26552, 10.8337]	0.09498 ± 0.01143	[0.06943, 0.12083]	0.0757	0.8633
A294	Arg-Fru	0.12107 ± 0.01324	[0.09156, 0.15058]	0.53345 ± 0.01156	[0.50769, 0.55921]	8.77e-04	0.9949
A294	Arg-Gal	0.19433 ± 0.03443	[0.11763, 0.27104]	1.26914 ± 0.03005	[1.20219, 1.3361]	0.0059	0.9939
A420	Arg-Fru	0.07553 ± 0.00901	[0.05547, 0.0956]	0.09158 ± 0.00786	[0.07407, 0.10909]	4.05e-04	0.9245
A420	Arg-Gal	0.21027 ± 0.02281	[0.15944, 0.26109]	0.25979 ± 0.01991	[0.21542, 0.30416]	0.0026	0.9390

Values are expressed as estimate ± SD. K represents the rate constant; A_0_ denotes the initial value. The 95% confidence intervals (CI) for A_0_ and K are shown in brackets. Adj. R^2^, adjusted coefficient of determination; reduced χ^2^, reduced chi-square. The selected models were zero-order for A294 and A420, and first-order for pH.

### 3.3 Substrate consumption and apparent melanoidin formation

**FIGURE 2:**
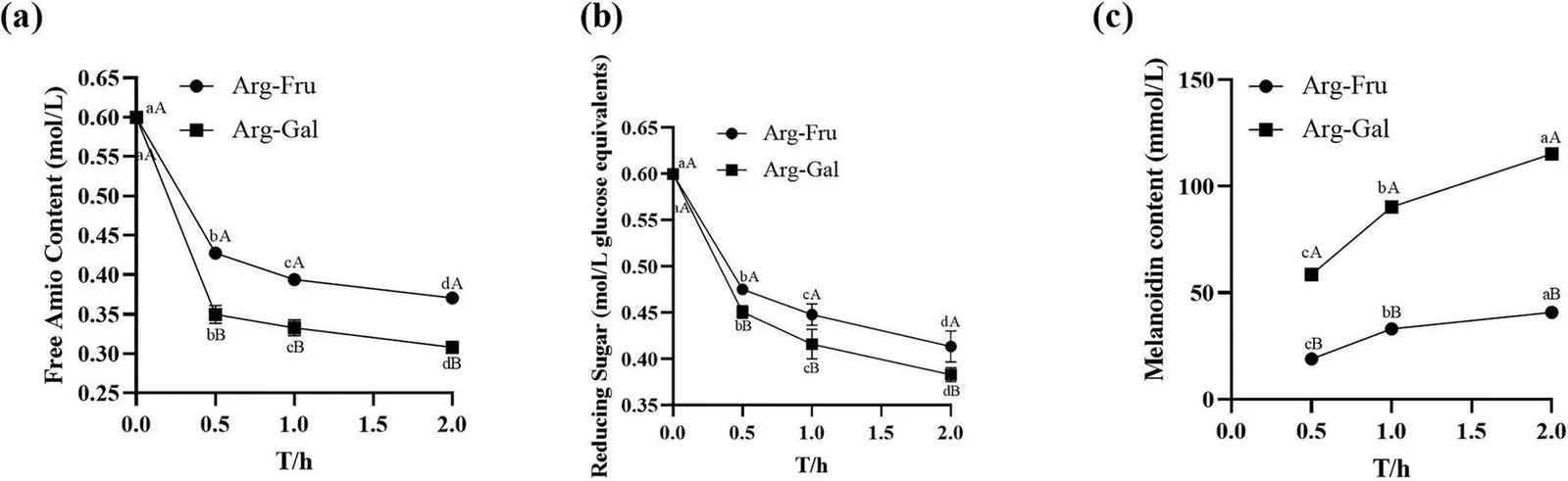
Free amino content of MRPs **(a)**; Reducing sugar content of MRPs **(b)**; Melanoidin content of MRPs **(c)**. Different lowercase letters indicate statistically significant differences (*P* < 0.05) among different time points within the same MRPs, while different uppercase letters denote significant differences (*P* < 0.05) between different MRPs at the same time point.

To further evaluate substrate consumption and melanoidin formation during the Maillard reaction, free amino groups, residual reducing sugars, and melanoidin content were analyzed, with the apparent melanoidin content estimated according to the Beer-Lambert law. The levels of free amino groups and residual reducing sugars are presented in Figures 2a,b, respectively. Both parameters decreased progressively with increasing heating time. Moreover, Arg-Gal consistently showed lower levels of free amino group and residual reducing sugar levels than Arg-Fru after 0.5, 1, and 2 h of heating. Furthermore, the apparent melanoidin content of the Arg-Gal was also significantly higher than that of the Arg-Fru (Figure 2c). These results indicate that, under the same reaction conditions, D-galactose exhibited higher reactivity toward L-arginine, as evidenced by greater substrate consumption and enhanced formation of melanoidin-like products.

### 3.4 Molecular docking analysis

**FIGURE 3:**
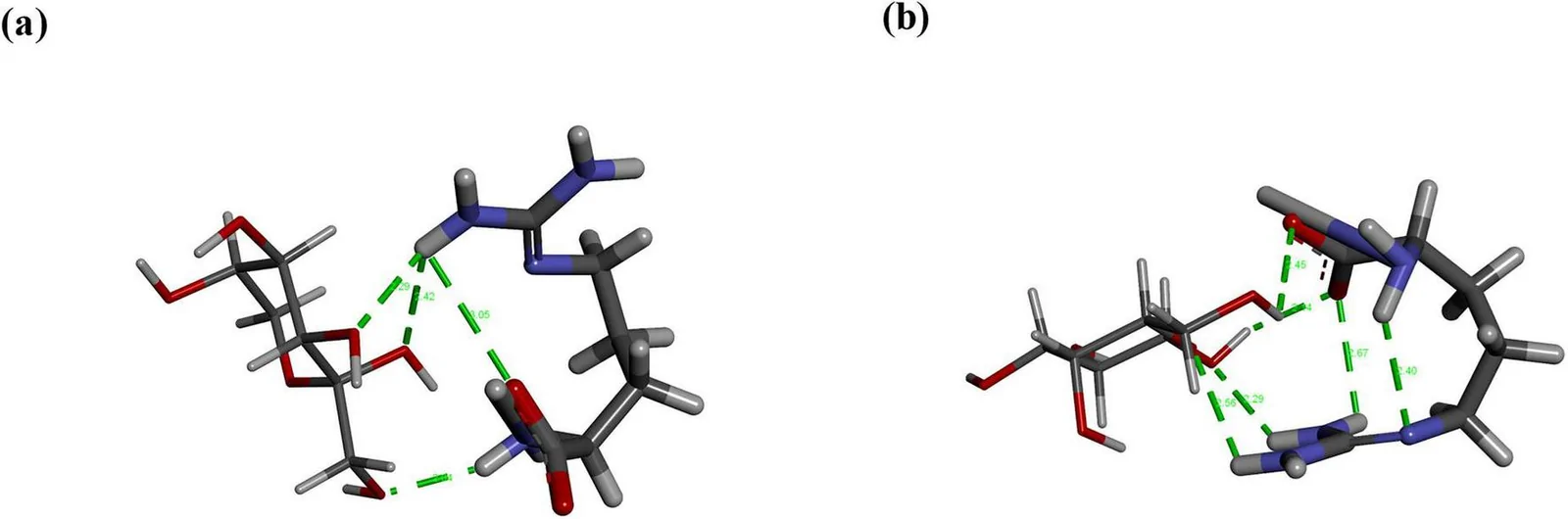
The docking results of D-fructose with L-arginine **(a)**; The docking results of D-galactose with L-arginine **(b)**.

As shown in Figure 3, the binding energy of the D-fructose and L-arginine complex was −1.8163 kcal/mol, and four hydrogen bonds were formed with bond lengths of 2.04, 2.29, 2.42, and 3.05 Å. In contrast, the binding energy between D-galactose and L-arginine was lower (−2.1493 kcal/mol), forming six hydrogen bonds with bond lengths of 2.14, 2.29, 2.40, 2.45, 2.56, and 2.67 Å. These results suggested that D-galactose may establish a more extensive hydrogen-bonding network with L-arginine than D-fructose.

### 3.5 Molecular dynamics simulation analysis

**FIGURE 4:**
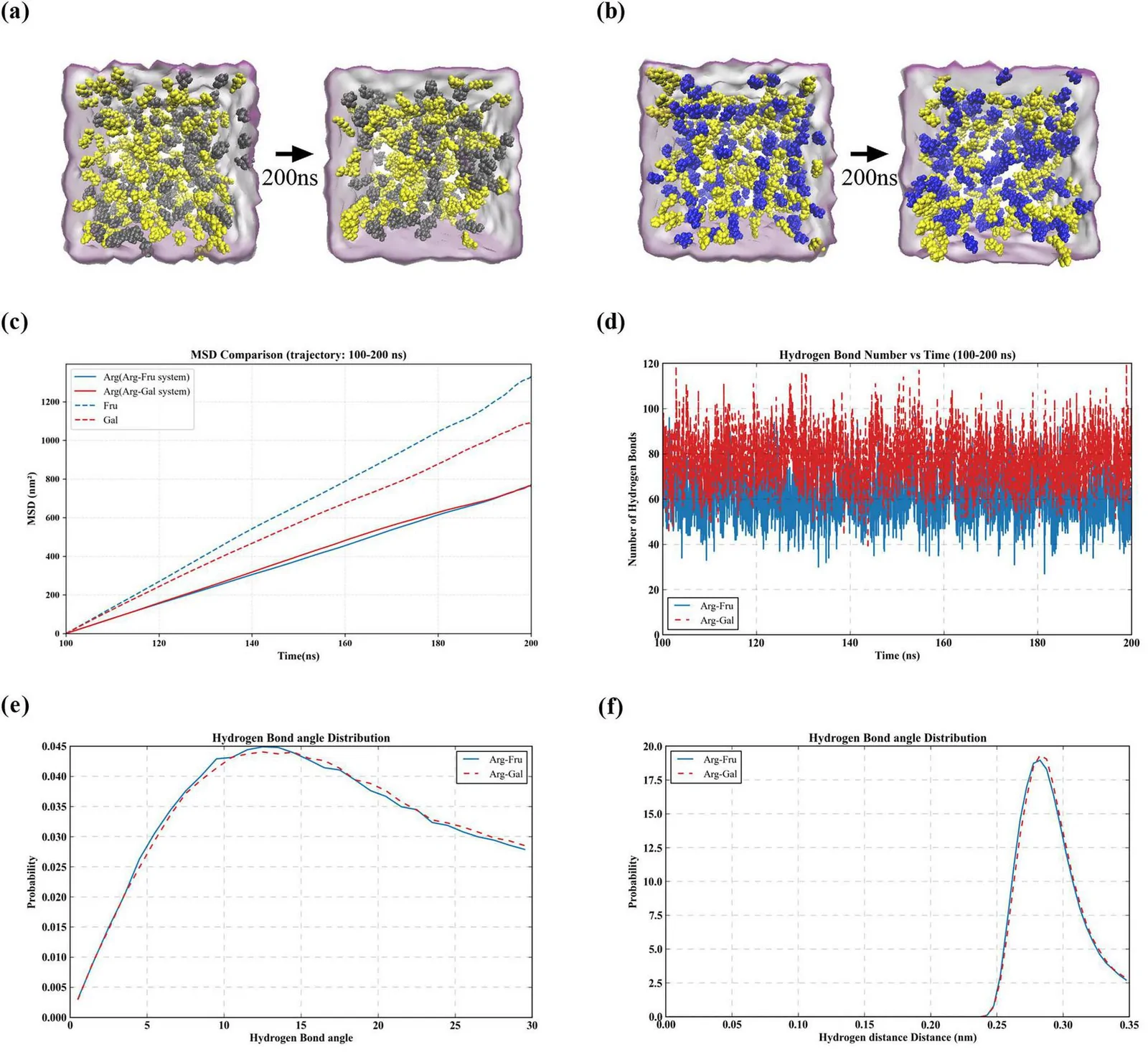
Structural diagrams of the Arg-Fru system before and after MD simulations **(a)**; Structural diagrams of the Arg-Gal system before and after MD simulations **(b)**; Mean square displacement (MSD) curves of molecular diffusion **(c)**; Number of hydrogen bonds between arginine and sugar molecules as a function of simulation time **(d)**; Hydrogen bond angle as a function of simulation time **(e)**; Hydrogen bond distance as a function of simulation time **(f)**.

All-atom MD simulations were performed to investigate the intermolecular interactions and diffusion behavior of arginine with D-fructose or D-galactose over a 200 ns trajectory (Figures 4a,b). Representative snapshots before and after the simulations showed that L-arginine and both monosaccharides were well dispersed in solution.

The mean square displacement (MSD) curves of the various molecules in the Maillard reaction systems are presented in Figure 4c, from which the corresponding diffusion coefficients were derived. During the entire simulation period, arginine exhibited similar diffusion in both systems, with MSD curves displaying good linearity over time and substantial overlap between the two systems. Accordingly, the diffusion coefficients were essentially identical, being 1.2743 × 10^−5^ cm^2^⋅s^−1^ in the fructose-containing system and 1.3270 × 10^−5^ cm^2^⋅s^−1^ in the galactose-containing system. In contrast, the MSD curves of monosaccharides increased more steeply, and their diffusion coefficients were higher than that of L-arginine, with values of 2.1233 × 10^−5^ cm^2^⋅s^−1^ for D-fructose and 1.7306 × 10^−5^ cm^2^⋅s^−1^ for D-galactose. It is worth noting that the MSD curve of D-galactose increased more gradually than that of D-fructose, and its diffusion coefficient was closer to that of L-arginine. This diffusion pattern suggests a potentially stronger non-covalent association between D-galactose and L-arginine.

To further examine this hypothesis, the number of hydrogen bonds formed between L-arginine and each monosaccharide was calculated from the MD trajectories. As shown in Figure 4d, the Arg-Gal system formed more hydrogen bonds than the Arg-Fru system. Specifically, the number of hydrogen bonds mainly fluctuated between 40 and 80 in the Arg-Fru system and between 60 and 100 in the Arg-Gal system. These results indicate that D-galactose can form more frequent hydrogen bonds with L-arginine, suggesting stronger intermolecular interaction between D-galactose and L-arginine. The hydrogen bond angle distributions were highly similar between the two systems, with both showing peaks around 12–13°, indicating comparable hydrogen bond geometry (Figure 4e). The hydrogen bond distance distribution shows that the bond lengths in both systems are concentrated within similar characteristic ranges, and the distribution curves highly overlap, indicating only minor differences in hydrogen-bond distance characteristics (Figure 4f). The corresponding uncertainty estimates for the diffusion coefficients and hydrogen-bond numbers, obtained by block-averaging analysis, are provided in Supplementary Table 2.

In summary, these results indicate that at 373.15 K (100°C), D-galactose shows stronger non-covalent association with L-arginine than D-fructose, as reflected by its lower diffusion coefficient and higher number of hydrogen bonds. This enhanced binding may increase the probability of effective molecular contact and potentially promote subsequent Maillard reactions.

### 3.6 Chemical antioxidant activity of MRPs

**FIGURE 5:**
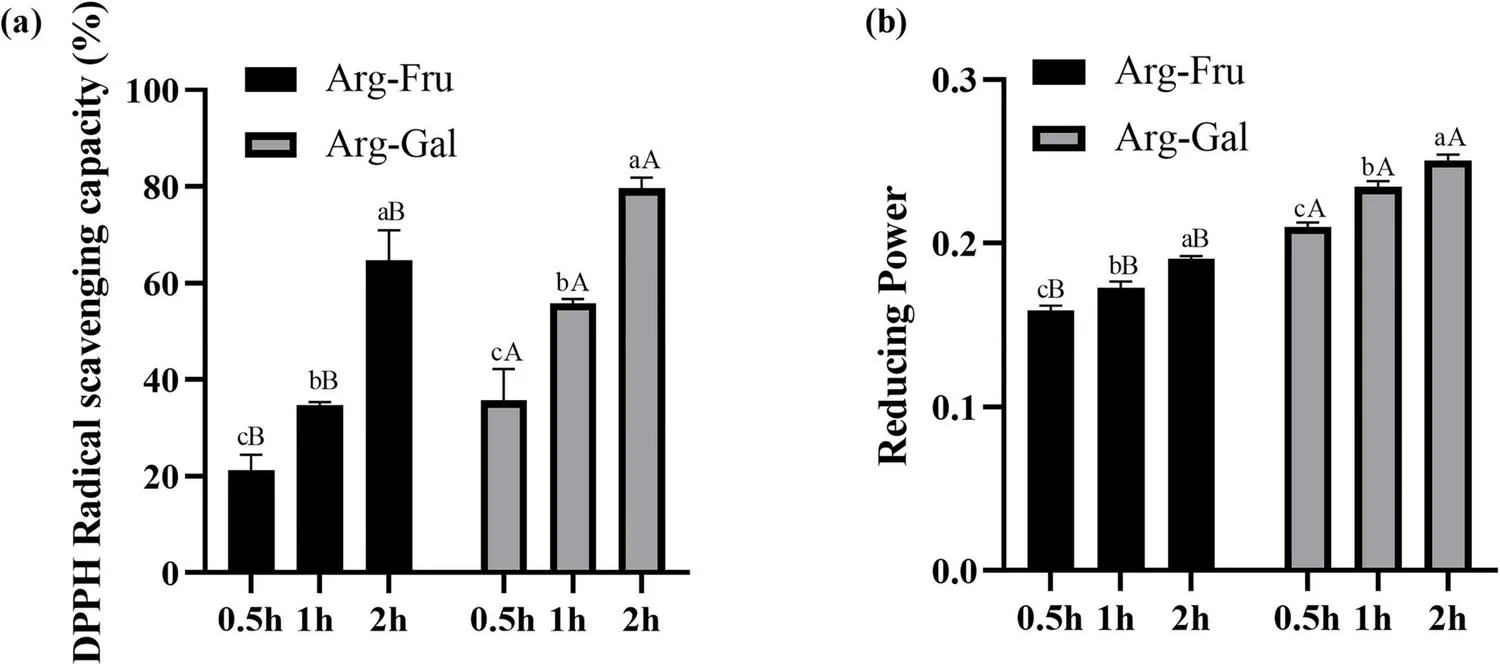
The DPPH radical scavenging activity of MRPs **(a)**; The reducing power of MRPs **(b)**. Different lowercase letters indicate statistically significant differences (*P* < 0.05) among different time points within the same MRPs, while different uppercase letters denote significant differences (*P* < 0.05) between different MRPs at the same time point.

The chemical antioxidant capacity of MRPs was evaluated by DPPH radical scavenging activity and reducing power. As shown in Figure 5a, DPPH radical scavenging activity gradually increased with heating time and reached the highest level at 2 h in both systems. The DPPH radical scavenging rate of the Arg-Fru ranged from 21.29% to 64.69%, and from 35.72% to 79.62% for Arg-Gal. The scavenging activity of the Arg-Gal was significantly higher than that of the Arg-Fru. Consistent with this trend, the absorbance at 700 nm (Figure 5b) increased continuously during heating, indicating a gradual enhancement in reducing power. Throughout the reaction, Arg-Gal consistently showed stronger reducing power than Arg-Fru. In addition, changes in reducing power closely paralleled those observed for DPPH radical scavenging activity. The chemical antioxidant capacities of the MRPs were further expressed as Vc equivalents (VCE, mmol/L) based on the corresponding Vc calibration curves, with the calculated VCE values and the results for Vc as the positive control summarized in Supplementary Tables 3, 4, respectively.

### 3.7 Cellular antioxidant activity of MRPs

**FIGURE 6:**
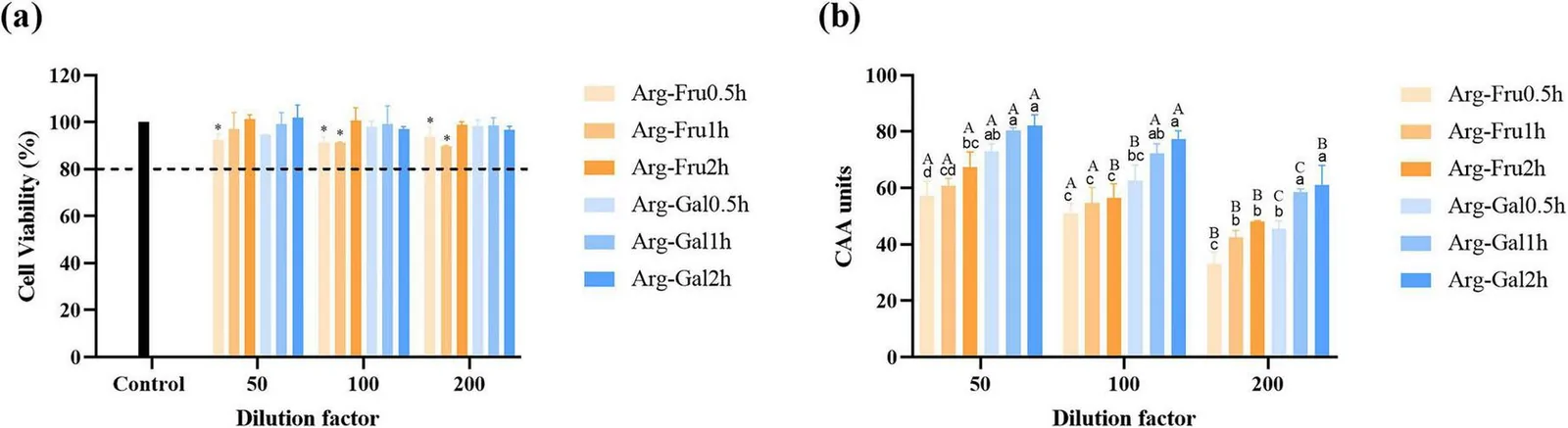
Viability effects of MRPs on RAW 264.7 cells **(a)**; Cellular antioxidant activity of MRPs **(b)**. *Indicates a significant difference (*P* < 0.05) compared with the control group; Different lowercase letters indicate significant differences among MRPs at the same dilution factor, whereas different uppercase letters indicate significant differences among dilution factors within the same MRPs (*P* < 0.05).

The effects of MRPs on RAW 264.7 cell viability are shown in Figure 6a. The results indicated that both types of MRPs had a limited effect on cell viability. For Arg-Fru samples, cell viability was significantly lower than that of the control at 0.5 h with dilution factors of 50, 100, and 200, reaching 92.34 ± 2.56%, 91.10 ± 2.53%, and 93.89 ± 4.06%, respectively, and at 1 h with dilution factors of 100 and 200, reaching 91.14 ± 0.54% and 89.83 ± 0.31%, respectively. However, cell viability remained above 80% in all treatment groups. Accordingly, the MRPs exhibited no marked cytotoxicity toward RAW 264.7 cells under the tested conditions.

As shown in Figure 6b, both types of MRPs exhibited intracellular antioxidant activity, with CAA units increasing as the dilution factor decreased. Overall, Arg-Gal exhibited stronger intracellular antioxidant capacity than Arg-Fru at all tested dilution factors. At 50-fold dilution, the CAA values of Arg-Gal after 0.5, 1, and 2 h of heating were 72.95 ± 2.64, 80.30 ± 1.10, and 82.07 ± 3.88, respectively, significantly higher than those of Arg-Fru (57.23 ± 5.16, 60.78 ± 2.54, and 67.52 ± 5.23). Similar trends were observed at 100-fold dilution, with CAA values of 62.64 ± 5.53, 72.40 ± 3.28, and 77.41 ± 2.85 for Arg-Gal, compared with 50.93 ± 3.61, 54.79 ± 5.38, and 56.39 ± 5.06 for Arg-Fru. At a dilution factor of 200, Arg-Gal also showed higher CAA values after 1 and 2 h of heating than Arg-Fru (58.44 ± 1.29 and 61.15 ± 6.87 vs. 42.55 ± 2.37 and 48.16 ± 0.32, respectively). The CAA of the MRPs was further expressed as quercetin equivalents (QE, μmol/L) based on the quercetin calibration curve, with the calculated QE values and the results for quercetin as the positive control summarized in Supplementary Tables 3, 4, respectively.

## 4 Discussion

By integrating experimental characterization with molecular simulations, this study systematically compared MRPs generated from L-arginine and two structurally distinct hexose reducing sugars, D-fructose and D-galactose, and investigated how sugar structure influences the reaction process, MRP formation, molecular interactions, and antioxidant activity. Previous studies comparing different reducing sugars in Maillard systems have mainly documented differences in browning development, product profiles, and biological activities (38, 39). Beyond these macroscopic comparisons, the present study further examined whether the distinct reaction behaviors of the Arg-Fru and Arg-Gal were associated with differences in their pre-reactive non-covalent interactions with L-arginine and related these molecular features to apparent reaction kinetics, substrate conversion, MRP formation, and antioxidant development.

Due to the formation of acidic compounds (such as formic acid and acetic acid) and the continuous consumption of amino compounds during the Maillard reaction, the pH of the arginine-based Maillard reaction system gradually decreased (40, 41). Compared to Arg-Fru, Arg-Gal consistently exhibited lower pH and higher absorbance at 294 nm and 420 nm, which may be related to the formation of intermediate products (such as furfural derivatives and reductones) and the relatively enhanced accumulation of melanoidins (42, 43). Kinetic analysis further showed higher apparent rate constants in the Arg-Gal system, suggesting greater Maillard reactivity under the same heating conditions. This finding is consistent with a previous study reporting greater browning and antioxidant activity in galactose- than fructose-based Maillard systems (44). However, sugar reactivity is substrate- and condition-dependent (16, 24); therefore, the higher reactivity of D-galactose observed here should be considered specific to the present L-arginine model. UV-Vis spectroscopy and fluorescence results also show that the fluorescence signals related to the conjugated chromophores and late glycosylation end products in the Arg-Gal are relatively stronger, suggesting that the Maillard reaction may have a deeper progression (45). Moreover, free amino groups and reducing sugars were depleted to a greater extent in the Arg-Gal, accompanied by significantly higher apparent melanoidin production, indicating more efficient substrate conversion and enhanced accumulation of melanoidin-related brown products (46, 47). The enhanced antioxidant activity of Arg-Gal was accompanied by increased formation of melanoidin-like products and other reaction-derived compounds, suggesting that multiple MRPs may collectively contribute to the overall antioxidant properties.

To further elucidate the molecular basis underlying the distinct reaction behaviors of the two model systems, molecular docking and MD simulations were performed to characterize the interactions between L-arginine and the two reducing sugars. Molecular docking analysis showed that D-galactose exhibited a slightly lower binding energy and formed more hydrogen bonds with L-arginine than D-fructose, suggesting a greater tendency to establish intermolecular interactions (48). Although the difference in binding energy was relatively small, the larger number of hydrogen bonds indicates that D-galactose may adopt a more favorable interaction pattern with L-arginine. This observation is consistent with the subsequent MD simulation results and suggests that structural differences between the two sugars may influence their non-covalent association with L-arginine before covalent bond formation. Such transient intermolecular interactions have been proposed to contribute to molecular recognition during the early stages of the Maillard reaction (20, 21). Therefore, the more extensive non-covalent interactions observed in the Arg-Gal system may represent one molecular factor contributing to the distinct reaction behaviors of the two model systems, rather than serving as the sole determinant of their reaction kinetics or antioxidant properties (49).

Molecular dynamics simulations further characterized the dynamic interactions between L-arginine and two reducing sugars in aqueous solution at 100 °C. Throughout the simulations, the Arg-Gal system formed more hydrogen bonds than the Arg-Fru system, with approximately 20 additional hydrogen bonds on average, indicating a denser hydrogen bond network. MSD analysis showed that D-galactose had a lower diffusion coefficient than D-fructose, and its diffusion behavior was closer to that of L-arginine. Although diffusion coefficients alone cannot demonstrate molecular binding, this diffusion pattern, together with the higher hydrogen-bond population, is consistent with a greater tendency for transient non-covalent association between D-galactose and L-arginine. Such an association may increase the probability of molecular encounters between D-galactose and L-arginine before subsequent reaction steps. Although the distributions of hydrogen-bond distances and angles were comparable between the two systems, the higher number of hydrogen bonds in the Arg-Gal suggests that the abundance and frequency of intermolecular contacts, rather than the geometry of individual hydrogen bonds, represent the major difference between the two systems (23, 48). This finding provides new mechanistic insight beyond previous comparisons of reducing sugars: the sugar-dependent difference in Maillard reactivity may be related not only to sugar classification or carbonyl structure but also to the population and dynamics of pre-reactive contacts formed with a specific amino acid. In summary, molecular docking and MD simulations suggest that structural differences between D-fructose and D-galactose may provide a molecular-level explanation for the different reaction behaviors observed in the two systems, with D-galactose forming a more extensively populated early non-covalent interaction network with L-arginine than D-fructose.

The Arg-Gal exhibited significantly higher DPPH radical scavenging activity, reducing power, and CAA than the Arg-Fru, indicating superior antioxidant properties. The antioxidant capacity of MRPs has been associated with various reaction-derived compounds, including reductones, nitrogen-containing heterocycles, melanoidin precursors, and other intermediates with redox activity (50, 51). Notably, the reducing power of the Arg-Gal after 1 h was comparable to that of the Arg-Fru after 2 h, suggesting earlier formation of electron-donating species (52). Combined with the spectroscopic profiles, substrate consumption behavior, and molecular simulation results, the more extensive pre-reactive association between D-galactose and L-arginine may facilitate early-stage Maillard reaction progression, promoting the generation of diverse reaction products and intermediates associated with antioxidant properties, thereby contributing to the improved radical scavenging and cellular antioxidant activities.

Since this study did not fully characterize the chemical structures of MRPs, the specific compounds leading to the observed biological activities remain unidentified. Furthermore, while molecular simulations provided valuable insights into early interactions, they represent an idealized model system and cannot fully capture the complexity of thermal Maillard reactions in real experimental settings. Therefore, future research should combine high-resolution mass spectrometry, chromatographic separation, structural identification, and targeted isolation of key intermediates to further validate the proposed mechanism.

Collectively, this study demonstrates that reducing sugar structure plays a critical role in regulating early non-covalent interactions with L-arginine, thereby affecting subsequent Maillard reaction progression, product formation, and functional properties of MRPs. Pearson correlation analysis, to some extent, illustrates the relationship between these indices (Supplementary Figures 4, 5). However, due to the limited number of model systems, quantitative relationships between molecular simulation descriptors and macroscopic experimental parameters could not be established. Nevertheless, the molecular docking and MD simulation results provided molecular-level evidence supporting a possible association between pre-reactive intermolecular organization and the distinct reactivity of the Arg-Gal and Arg-Fru systems. Overall, these findings highlight that the reaction behavior of a reducing sugar toward a specific amino acid may depend not only on its general structural classification but also on substrate-pair-specific molecular recognition and dynamic association in solution.

## 5 Conclusion

Compared with D-fructose, D-galactose showed higher Maillard reactivity with L-arginine under the same heating conditions, leading to greater substrate consumption and enhanced formation of MRPs. Molecular docking and MD simulations suggested stronger non-covalent association between D-galactose and L-arginine, characterized by a denser hydrogen-bonding network and a diffusion behavior closer to that of L-arginine. These molecular features may increase the probability of early-stage molecular encounters and contribute to reaction progression. Correspondingly, Arg-Gal MRPs exhibited stronger DPPH radical scavenging activity, reducing power, and cellular antioxidant capacity than Arg-Fru MRPs. These findings highlight the important role of sugar structure in modulating the reactivity and functional properties of arginine-derived Maillard reaction systems.

## Data Availability

The original contributions presented in this study are included in this article/Supplementary material, further inquiries can be directed to the corresponding author.
