# Supplementary material for: D-galactose enhances Maillard reaction and antioxidant activity of arginine-derived products: experimental and molecular insights
Source: Front Nutr. 2026 Aug 28;13:1924087. doi: 10.3389/fnut.2026.1924087 (PMC13562050; doi:10.3389/fnut.2026.1924087)
Supplement: Supplementary file 1 [file Data_Sheet_1.pdf]

Supplementary material

1. Model selection and statistical validation

Kinetic models were selected using a multi-criteria framework rather than the coefficient of determination (R^2) alone. The criteria included (i) adjusted R^2, which accounts for model complexity; (ii) reduced chi-squar, for which lower values indicate smaller discrepancies between observed and fitted values; (iii) 95% confidence intervals (CIs) for the fitted rate constants; and (iv) four-panel residual diagnostics.

Table S1. Kinetic model fitting parameters for different experimental groups

Parameter	Group	Model	A_0	A_0 95%CI	K	K 95%CI	Reduced χ^2	Adj.R^2
pH	Arg-Fru	Zero-order	9.75833±0.19508	[9.32367,10.19300]	1.00e-14±0.00e+00	[1.00e-14,1.00e-14]	0.4567	-0.1000
pH	Arg-Fru	First-order	10.44642±0.12533	[10.16673,10.7277]	0.07988±0.01124	[0.05476,0.10527]	0.0746	0.8204
pH	Arg-Gal	Zero-order	10.51333±0.13068	[10.22217,10.8045]	-0.89333±0.11406	[-1.14748,-0.63919]	0.0854	0.8458
pH	Arg-Gal	First-order	10.54881±0.12687	[10.26552,10.8337]	0.09498±0.01143	[0.06943,0.12083]	0.0757	0.8633
A294	Arg-Fru	Zero-order	0.12107±0.01324	[0.09156,0.15058]	0.53345±0.01156	[0.50769,0.55921]	8.77e-04	0.9949
A294	Arg-Fru	First-order	0.25879±0.03784	[0.1777,0.34272]	-0.76691±0.08319	[-0.95675,-0.60248]	0.0140	0.9180
A294	Arg-Gal	Zero-order	0.19433±0.03443	[0.11763,0.27104]	1.26914±0.03005	[1.20219,1.33610]	0.0059	0.9939
A294	Arg-Gal	First-order	0.54417±0.09281	[0.36038,0.75000]	-0.81117±0.09592	[-1.03258,-0.62521]	0.0883	0.9086
A420	Arg-Fru	Zero-order	0.07553±0.00901	[0.05547,0.09560]	0.09158±0.00786	[0.07407,0.10909]	4.05e-04	0.9245
A420	Arg-Fru	First-order	0.09405±0.01074	[0.07189,0.11776]	-0.50284±0.01741	[-0.66093,-0.35625]	8.62e-04	0.8396
A420	Arg-Gal	Zero-order	0.21027±0.02281	[0.15944,0.26109]	0.25979±0.01991	[0.21542,0.30416]	0.0026	0.9390
A420	Arg-Gal	First-order	0.26384±0.02946	[0.20306,0.32879]	-0.50490±0.06976	[-0.65898,-0.36181]	0.0065	0.8476

Note:Values are presented as estimate ± SD (n = 3).

For intermediate-product formation (A294) and brown-pigment development (A420), the zero-order model provided the best overall fit in both systems, with adjusted R^2 values above 0.92 and reduced χ^2 values below 0.006. For pH, the first-order model was selected using the same assessment framework. The residuals of the selected models were randomly distributed without evident systematic patterns, supporting the adequacy of the fitted models.

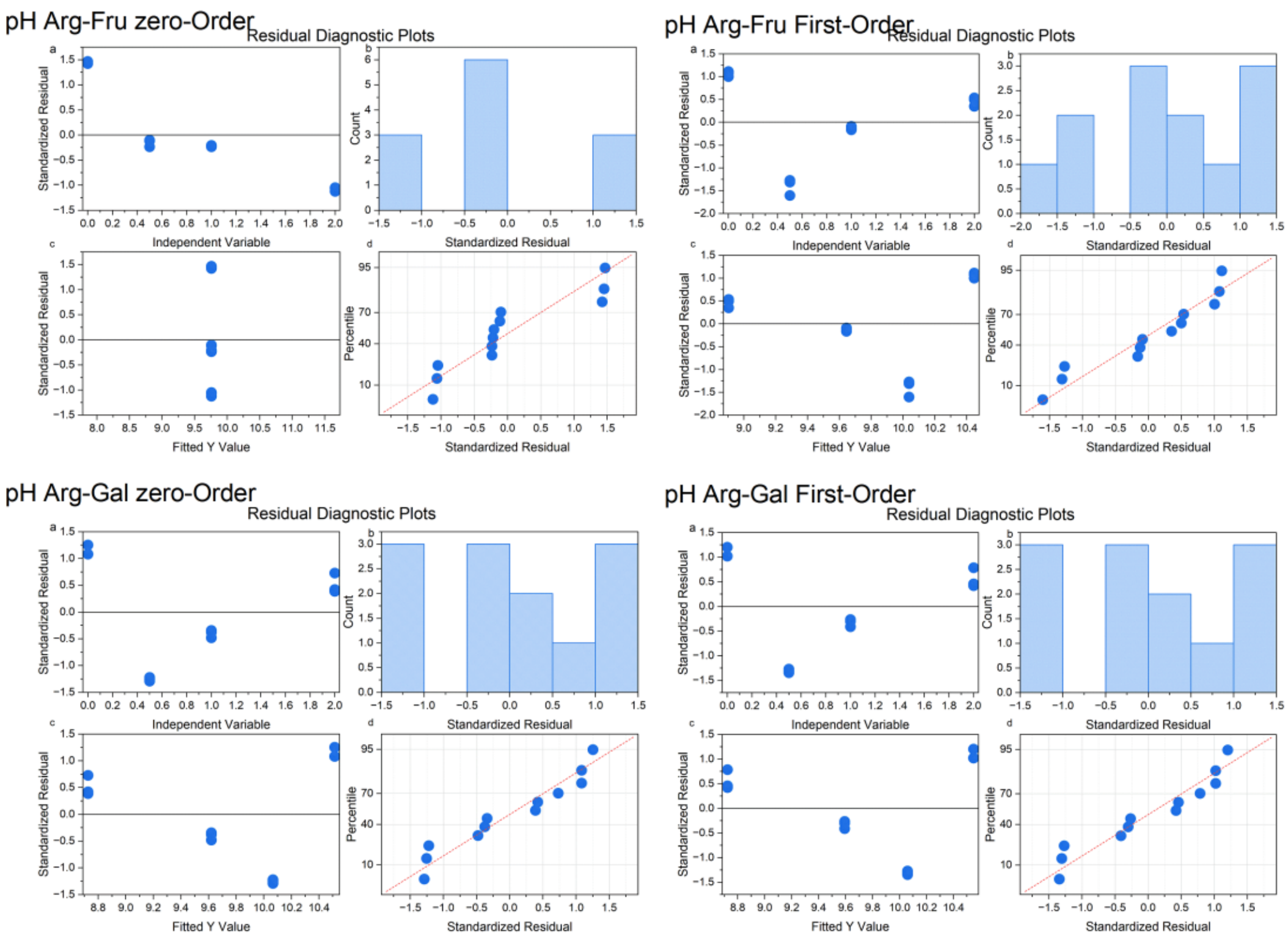

Figure S1. Residual diagnostic plots for pH fitted kinetic models: (a) standardized residuals versus independent variable; (b) histogram of standardized residuals; (c) standardized residuals versus fitted values; and (d) normal probability (Q-Q) plot.

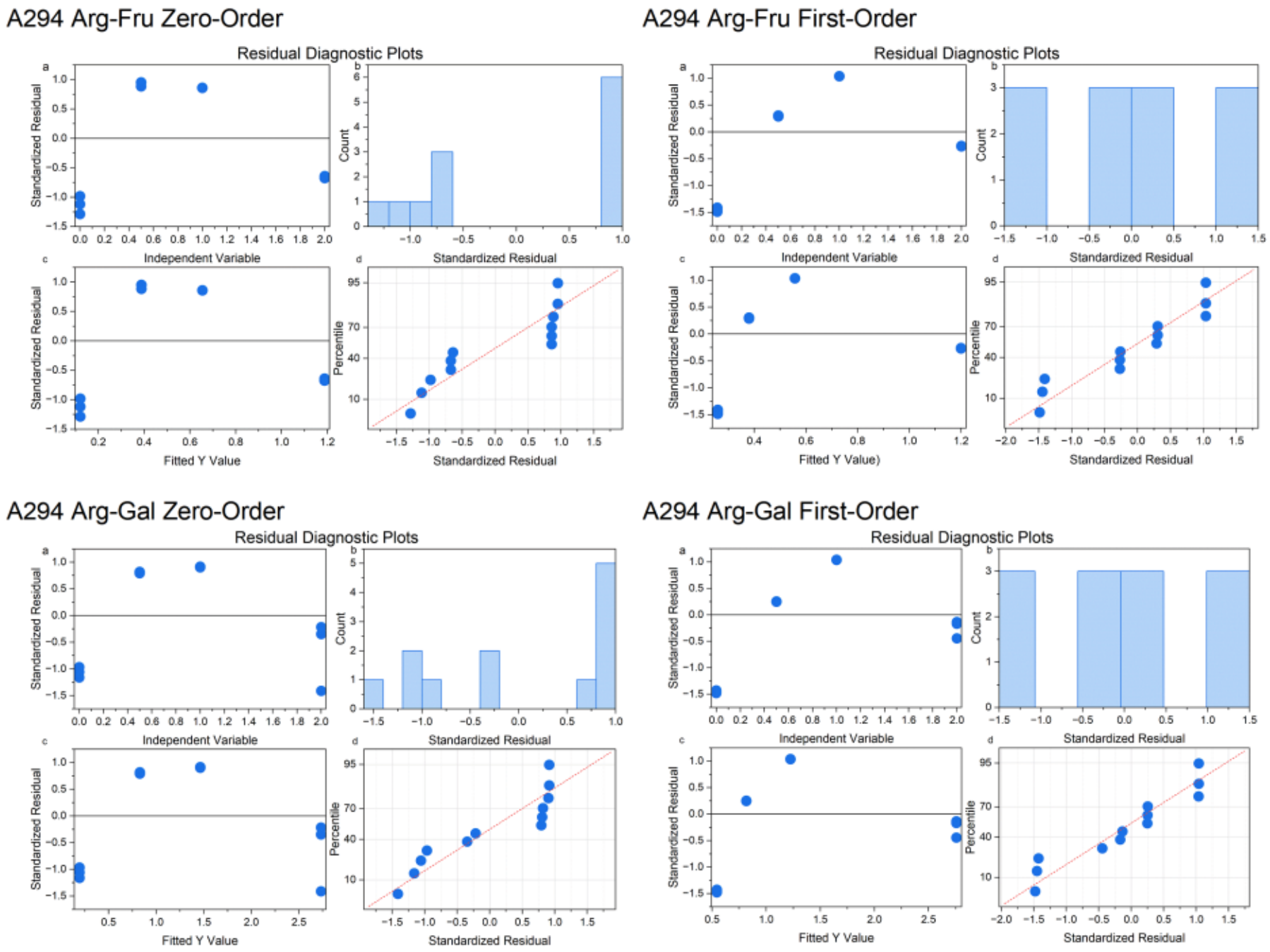

Figure S2. Residual diagnostic plots for A294 fitted kinetic models: (a) standardized residuals versus independent variable; (b) histogram of standardized residuals; (c) standardized residuals versus fitted values; and (d) normal probability (Q-Q) plot.

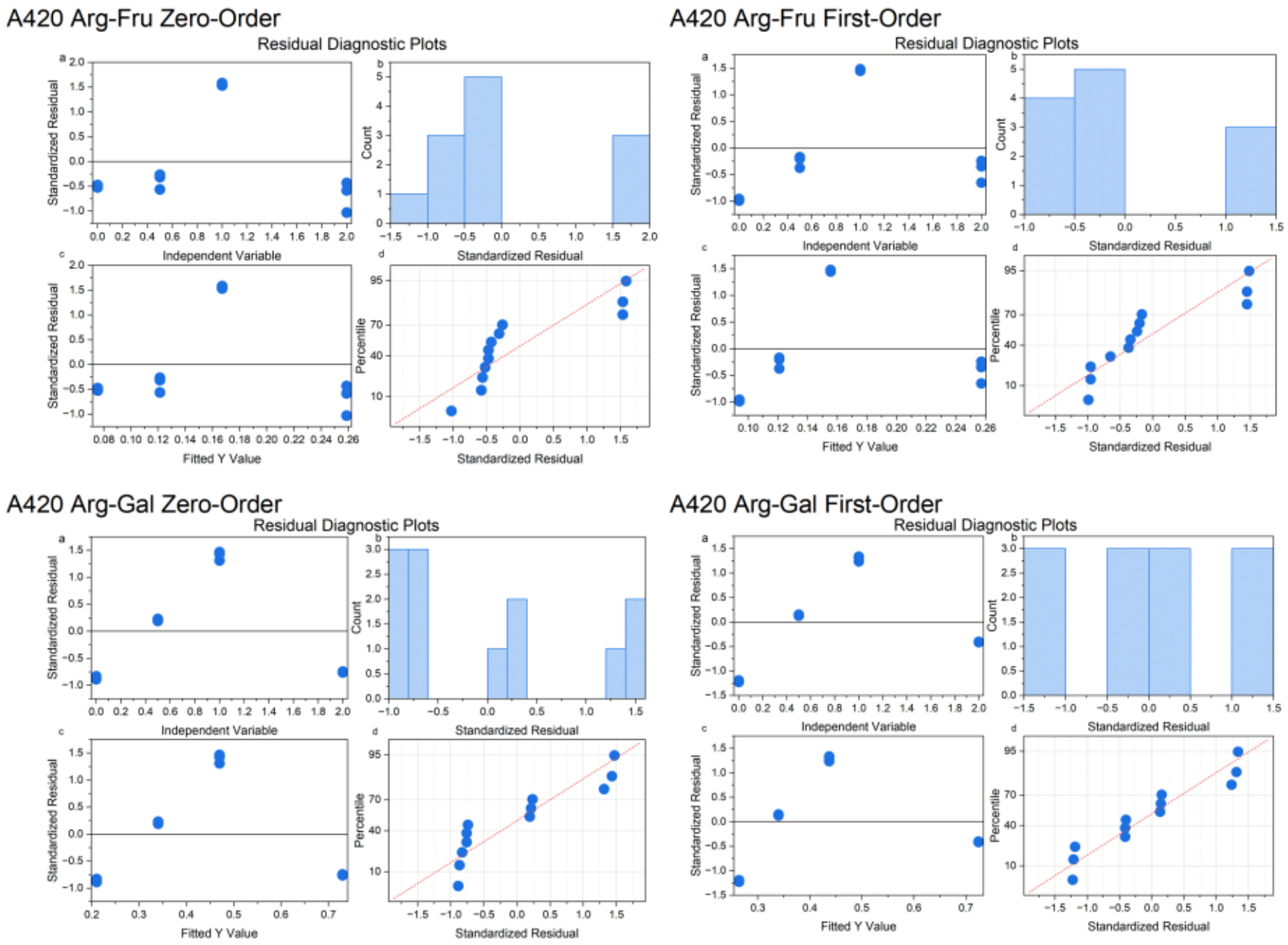

Figure S3. Residual diagnostic plots for A420 fitted kinetic models: (a) standardized residuals versus independent variable; (b) histogram of standardized residuals; (c) standardized residuals versus fitted values; and (d) normal probability (Q-Q) plot.

2. Uncertainty estimation of Trajectory-Derived Parameters

Uncertainty was estimated by dividing the equilibrated trajectory segment (100-200 ns) into four equal, non-overlapping blocks. Hydrogen-bond numbers and diffusion coefficients were calculated independently for each block and are reported as the mean ± SD of the block-derived values

Table S2. Block-averaged uncertainty estimates for hydrogen-bond numbers and diffusion coefficients obtained from the equilibrated MD trajectorie

Parameter	Molecule/system	Mean ± SD
Hydrogen-bond number	Arg-Fru	61.2037 ± 0.9819
Hydrogen-bond number	Arg-Gal	77.6862 ± 0.5094
Diffusion coefficient ($\times 10^{-5}$ cm^2 s^{-1})	Arg (Arg-Fru)	1.2712 ± 0.0343
Diffusion coefficient ($\times 10^{-5}$ cm^2 s^{-1})	Arg (Arg-Gal)	1.2723 ± 0.1110
Diffusion coefficient ($\times 10^{-5}$ cm^2 s^{-1})	Fru (Arg-Fru)	2.2143 ± 0.0953
Diffusion coefficient ($\times 10^{-5}$ cm^2 s^{-1})	Gal (Arg-Gal)	1.8256 ± 0.1643

Note: The equilibrated trajectory segment was divided into four equal, non-overlapping blocks. Each parameter was calculated independently for every block using GROMACS analysis tools; values are reported as mean ± SD across the four blocks.

3. Antioxidant Capacity Expressed

Calibration curves and equivalent-expression procedures

DPPH radical-scavenging activity: Vitamin C standards (0.0025, 0.005, 0.00625, 0.0125, 0.025, and 0.05 mM) were used to establish the calibration curve for DPPH radical scavenging capacity: y=1580x+5.0122 (R^2=0.9933). The DPPH radical scavenging capacity of MRPs was expressed as vitamin C equivalents (VCE, mmol/L).

Reducing power: Vitamin C standards (0, 0.005, 0.00625, 0.0125, 0.025, 0.05, and 0.1 mM) were used to establish the calibration curve for reducing power: y=1.7211x+0.0643 (R^2=0.9962). The reducing power of MRPs was expressed as vitamin C equivalents (VCE, mmol/L).

Cellular antioxidant activity: Quercetin standards (2, 4, 6, 8, 10 μM) were used to establish the calibration curve for cellular antioxidant activity: y=5.6938x+5.9265 (R^2=0.9957). The cellular antioxidant activity of MRPs was expressed as Quercetin equivalents (QE, mmol/L).

Table S3. DPPH radical-scavenging capacity, reducing power, and cellular antioxidant activity of MRPs expressed as reference-compound equivalents

Parameter	0.5h Arg-Fru	1h Arg-Fru	2h Arg-Fru	0.5h Arg-Gal	1h Arg-Gal	2h Arg-Gal
DPPH (mmol VCE/L)	1.03 ± 0.20^{cB}	1.87 ± 0.04^{bB}	3.77 ± 0.39^{aB}	1.94 ± 0.41^{cA}	3.21 ± 0.05^{bA}	4.72 ± 0.14^{aA}
Reducing power (mmol VCE/L)	27.51 ± 0.82^{cB}	32.83 ± 1.04^{bB}	36.71 ± 0.44^{aB}	42.32 ± 0.76^{cA}	49.39 ± 1.02^{bA}	54.14 ± 1.02^{aA}
CAA (mM QE/L)	0.79 ± 0.06^{aB}	0.85 ± 0.09^{aB}	0.88 ± 0.08^{aB}	0.99 ± 0.07^{bA}	1.16 ± 0.05^{abA}	1.25 ± 0.05^{aA}

Note: Values are presented as mean ± standard deviation (n = 3). Different lowercase letters indicate significant differences among MRPs with different heating times, while different uppercase letters indicate significant differences between different MRPs at the same heating time ($p < 0.05$).

Antioxidant positive control

Table S4. Antioxidant activities of vitamin C and quercetin used as positive controls

Positive control	Concentration	DPPH radical scavenging activity (%)	Reducing power (A700)	CAA units
Vitamin C	0.05 mM	82.94±2.45	——	——
Vitamin C	0.10 mM	——	0.23±0.04	——
Quercetin	10 μM	——	——	62.06±5.68

Note: Values are presented as mean ± SD (n = 3). A700 denotes absorbance at 700 nm.

4. Correlation analysis

Pearson correlation analysis was conducted separately for the Arg-Fru and Arg-Gal systems using experimental data collected at 0, 0.5, 1, and 2 h. The variables included pH, A294, A420, free amino-group content, residual reducing-sugar content, apparent melanoidin content, reducing power, DPPH radical-scavenging activity, and cellular antioxidant activity (CAA). The resulting correlation matrices are shown in Figures S4 and S5. Because each system contained only four time points, the correlations were used descriptively to visualize coordinated trends and were not interpreted as evidence of causality.

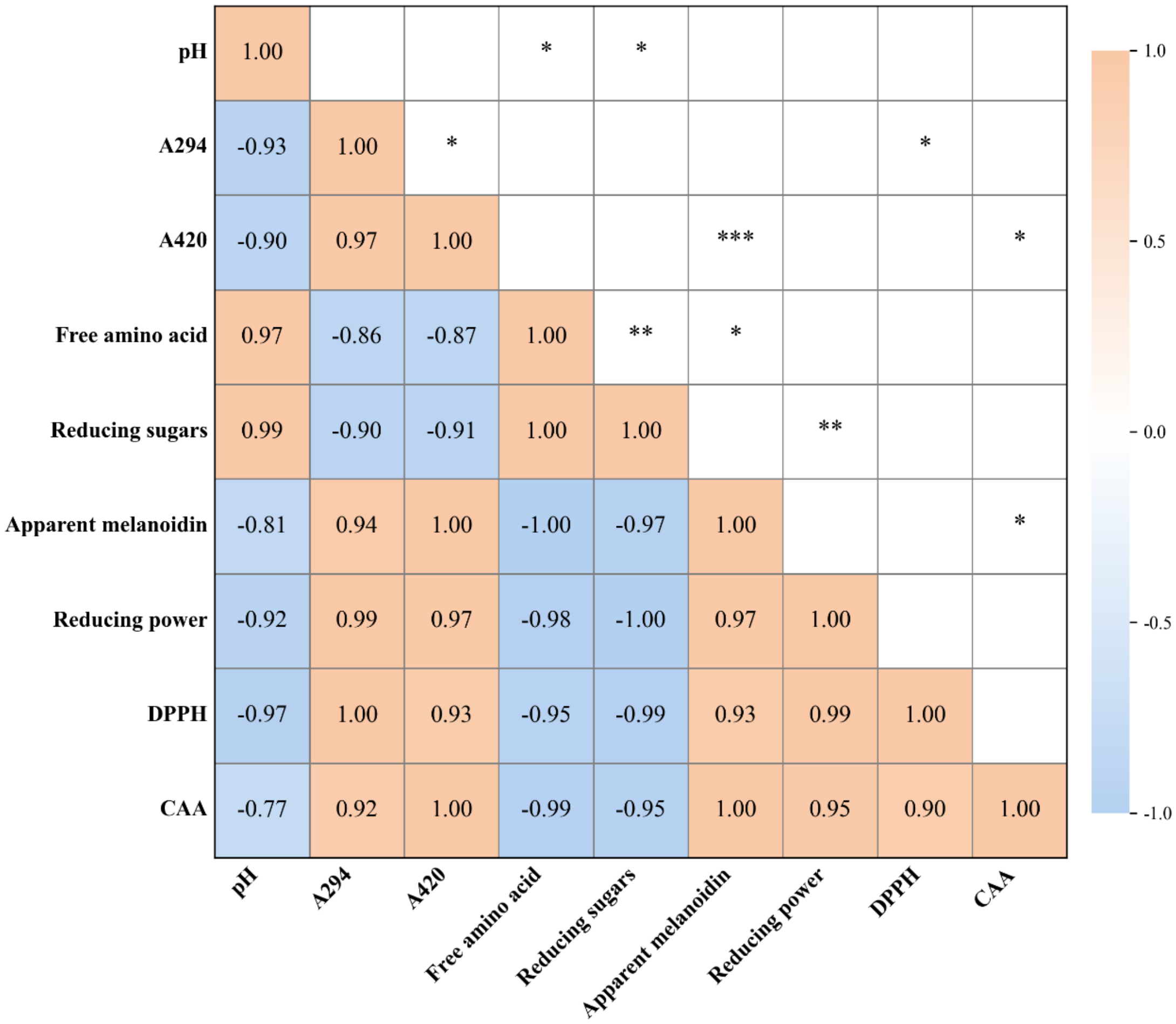

Figure S4. Pearson correlation analysis between reaction progress indicators and bioactivity parameters of Arg-Fru. Pearson correlation coefficients (r) are shown in the cells. Color intensity represents the magnitude and direction of the correlations. Asterisks indicate statistically significant correlations: *P < 0.05, **P < 0.01, ***P < 0.001.

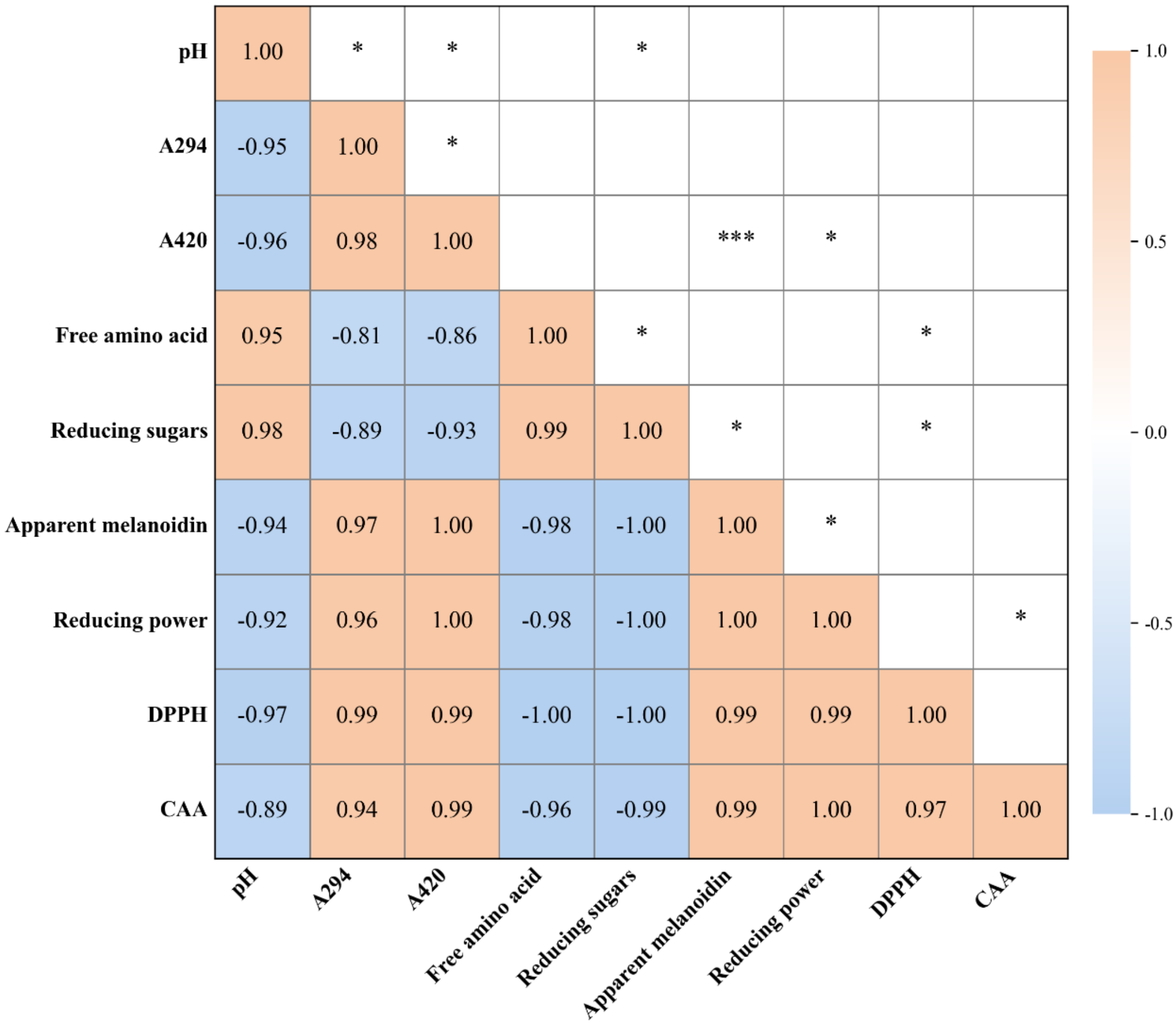

Figure S5. Pearson correlation analysis between reaction progress indicators and bioactivity parameters of Arg-Gal. Pearson correlation coefficients (r) are shown in the cells. Color intensity represents the magnitude and direction of the correlations. Asterisks indicate statistically significant correlations: *P < 0.05, **P < 0.01, ***P < 0.001.
